# p38 mitogen-activated protein kinase and mitogen-activated protein kinase-activated protein kinase 2 (MK2) signaling in atrophic and hypertrophic denervated mouse skeletal muscle

**DOI:** 10.1186/1750-2187-9-2

**Published:** 2014-03-15

**Authors:** Kim Evertsson, Ann-Kristin Fjällström, Marlene Norrby, Sven Tågerud

**Affiliations:** 1 Department of Chemistry and Biomedical Sciences, Linnaeus University, SE-391 82 Kalmar, Sweden

**Keywords:** Denervation, Hsp25, Hsp70, MK2, p38, Skeletal muscle, Phosphorylation, Cytosolic, Nuclear fractions

## Abstract

**Background:**

p38 mitogen-activated protein kinase has been implicated in both skeletal muscle atrophy and hypertrophy. T317 phosphorylation of the p38 substrate mitogen-activated protein kinase-activated protein kinase 2 (MK2) correlates with muscle weight in atrophic and hypertrophic denervated muscle and may influence the nuclear and cytoplasmic distribution of p38 and/or MK2. The present study investigates expression and phosphorylation of p38, MK2 and related proteins in cytosolic and nuclear fractions from atrophic and hypertrophic 6-days denervated skeletal muscles compared to innervated controls.

**Methods:**

Expression and phosphorylation of p38, MK2, Hsp25 (heat shock protein25_rodent_/27_human_, Hsp25/27) and Hsp70 protein expression were studied semi-quantitatively using Western blots with separated nuclear and cytosolic fractions from innervated and denervated hypertrophic hemidiaphragm and atrophic anterior tibial muscles. Unfractionated innervated and denervated atrophic pooled gastrocnemius and soleus muscles were also studied.

**Results:**

No support was obtained for a differential nuclear/cytosolic localization of p38 or MK2 in denervated hypertrophic and atrophic muscle. The differential effect of denervation on T317 phosphorylation of MK2 in denervated hypertrophic and atrophic muscle was not reflected in p38 phosphorylation nor in the phosphorylation of the MK2 substrate Hsp25. Hsp25 phosphorylation increased 3-30-fold in all denervated muscles studied. The expression of Hsp70 increased 3-5-fold only in denervated hypertrophic muscles.

**Conclusions:**

The study confirms a differential response of MK2 T317 phosphorylation in denervated hypertrophic and atrophic muscles and suggests that Hsp70 may be important for this. Increased Hsp25 phosphorylation in all denervated muscles studied indicates a role for factors other than MK2 in the regulation of this phosphorylation.

## Background

It is likely that skeletal muscle atrophy (muscle wasting) and hypertrophy (muscle growth) are caused by alterations in the balance between protein synthesis and degradation. It has been suggested that one pathway which plays a role in this balance is the p38 mitogen-activated protein kinase (p38 MAPK, p38) pathway [1]. p38 has been identified as a likely mediator of catabolic signaling in skeletal muscle [2]. Thus, inhibitors of p38 block TNF-α induced expression of the E3 ubiquitin ligases atrogin1/muscle atrophy F-box (MAFbx) [3] and muscle-specific ring finger protein 1 (MuRF1) [4] as well as lipopolysaccharide (LPS) stimulated atrogin1/MAFbx expression and loss of muscle mass [5]. Increased p38 activation (phosphorylation) has also been reported in models of muscle inactivity such as cast immobilization [6], denervation [7] and hind-limb unloading [8]. However, p38 has also been associated with muscle growth since p38 activation (phosphorylation) occurs in response to functional overload and mechanical stimuli such as stretch and exercise [9-14].

In vertebrates four isoforms of p38 have been identified: p38α (MAPK14 also called CSAIDs binding protein (CSBP) and SAPK2a), p38β (MAPK11 also called SAPK2b and p38-2), p38γ (MAPK12 also called SAPK3 and ERK6) and p38δ, (MAPK13 also called SAPK4) [15]. Activation of the p38 isoforms occurs by phosphorylation of a conserved threonine-glycine-tyrosine (TGY) dual phosphorylation motif by upstream kinases MKK3 (mitogen-activated protein kinase kinase 3, MAP2K3), MKK6 (mitogen-activated protein kinase kinase 6, MAP2K6) and MKK4 (mitogen-activated protein kinase kinase 4, MAP2K4) [16,17]. Once activated, p38 isoforms phosphorylate serine/threonine residues in their substrates [18].

One substrate of p38 is mitogen-activated protein kinase-activated protein kinase 2 (MAPKAPK2, MK2) with proposed roles in actin remodeling, cell migration, development and regulation of the cell cycle [19]. In the mouse, p38 phosphorylates MK2 on two sites, T205 and T317 (correspond to T222 and T334 in the human sequence) [20,21]. Both sites are considered important for activation of MK2. Phosphorylation on T317 may also serve as a switch for MK2 nuclear import and export [22]. When MK2 becomes phosphorylated on T317 the autoinhibitory helix is released from the core of the kinase domain thereby exposing the nuclear export signal, which allows MK2 to leave the nucleus in a complex with p38 [23,24]. It has, however, also been suggested that MK2 can exit the nucleus without p38 but still dependent upon its phosphorylation [25]. In the nucleus MK2 contributes to the phosphorylation of transcription factors such as cAMP response element-binding protein (CREB) [26] and serum response factor (SRF) [27]. In the cytoplasm, MK2 can phosphorylate a small heat shock protein (Hsp25_rodent_/27_human_) [28], suggested to play crucial roles in the regulation of the actin cytoskeleton [29]. Phosphorylation of Hsp25/27 has been shown to increase in skeletal muscle hypertrophy and decrease in atrophy [13,30]. Overexpression of Hsp27 also inhibits disuse induced MuRF1 and atrogin1/MAFbx transcription and muscle fiber atrophy [31].

Hsp70, another heat shock protein that has been shown to prevent skeletal muscle atrophy when overexpressed [32,33], was recently reported to play a role in the phosphorylation of MK2 by p38 [34]. Hsp70 has been shown to be down-regulated in atrophic conditions [35-38] and to be up-regulated in hypertrophic conditions [39-44].

Denervation of skeletal muscles generally causes atrophy of the muscles but the hemidiaphragm becomes transiently hypertrophic following denervation [45-48]. We have previously shown that the MK2 phosphorylation on T317 correlates with muscle weight in denervated atrophic anterior tibial muscle, denervated hypertrophic hemidiaphragm muscle and innervated control muscles [48]. Since the T317 phosphorylation is likely to be important for nuclear export of MK2, possibly in complex with p38, the previous study suggested that in hypertrophic denervated muscle MK2 and/or p38 may be localized mainly in the cytoplasm whereas in denervated atrophic muscle they may be localized in the nucleus to a larger extent. The present study examines the hypothesis that the differential response of hemidiaphragm and hind-limb muscles to denervation is related to a differential nuclear/cytosolic localization of p38 and/or MK2. Separated cytosolic and nuclear fractions were prepared from 6-days denervated hemidiaphragm muscle (hypertrophic) and 6-days denervated anterior tibial muscle (atrophic). The protein expression and phosphorylation status of p38, MK2 and Hsp25 as well as Hsp70 protein expression were examined in the fractions. The mouse anterior tibial muscle is devoid of type I muscle fibers [49] whereas the hemidiaphragm of the mouse contains about 12% of type I fibers [50]. The proteins mentioned (p38, MK2, Hsp25 and Hsp70) were, therefore, also examined in unfractionated homogenates of pooled gastrocnemius and soleus muscles in order to examine hind-limb muscles that, like the hemidiaphragm, contain type I muscle fibers in addition to type II fibers [50,51] but like the anterior tibial muscle become atrophic following denervation.

## Results

All results reported are based on data from 4 sets of 8 animals generating 8 denervated anterior tibial muscles with 8 contralateral innervated controls, 8 denervated pooled gastrocnemius and soleus muscles with 8 contralateral innervated controls, 8 denervated hemidiaphragm muscles and 8 innervated control hemidiaphragms from separate animals.

### Muscle weights

Six days after denervation hemidiaphragm muscles were hypertrophic with a wet weight of 40.3 ± 1.5 mg compared to 29.6 ± 0.6 mg for innervated controls (p < 0.001, Student’s t-test). Six days after denervation anterior tibial muscles were atrophic with a wet weight of 44.7 ± 1.8 mg compared to 60.8 ± 1.9 mg for innervated controls (p < 0.001, Student’s t-test). Six days after denervation pooled gastrocnemius and soleus muscles were atrophic with a wet weight of 149.1 ± 4.4 mg, compared to innervated controls with a wet weight of 198.3 ± 5.7 mg (p < 0.001, Student’s paired t-test).

### Markers of nuclear and cytosolic fractions

**Figure 1:**
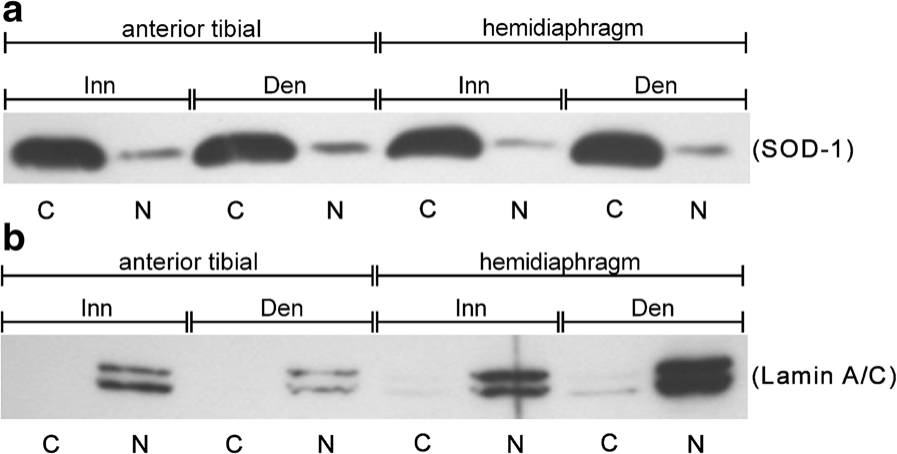
**Separation of nuclear and cytosolic fractions.** Western blots with SOD-1 (**a**, cytosolic marker) and Lamin A/C (**b**, nuclear marker) demonstrating separation of cytosolic (C) and nuclear (N) fractions. Lanes from left to right contain cytosolic and nuclear fractions from: innervated (Inn) anterior tibial muscle, 6-days denervated (Den) anterior tibial muscle, innervated hemidiaphragm and 6-days denervated hemidiaphragm.

Successful separation of nuclear and cytosolic fractions was confirmed using Western blots for Lamin A/C and superoxide dismutase 1 (SOD-1), respectively. Very little nuclear protein (Lamin A/C) was present in cytosolic fractions and only a small amount of cytosolic protein (SOD-1) was present in the nuclear fraction (Figure 1).

### Protein expression in nuclear and cytosolic fractions of 6-days denervated hypertrophic hemidiaphragm muscle

**Figure 2:**
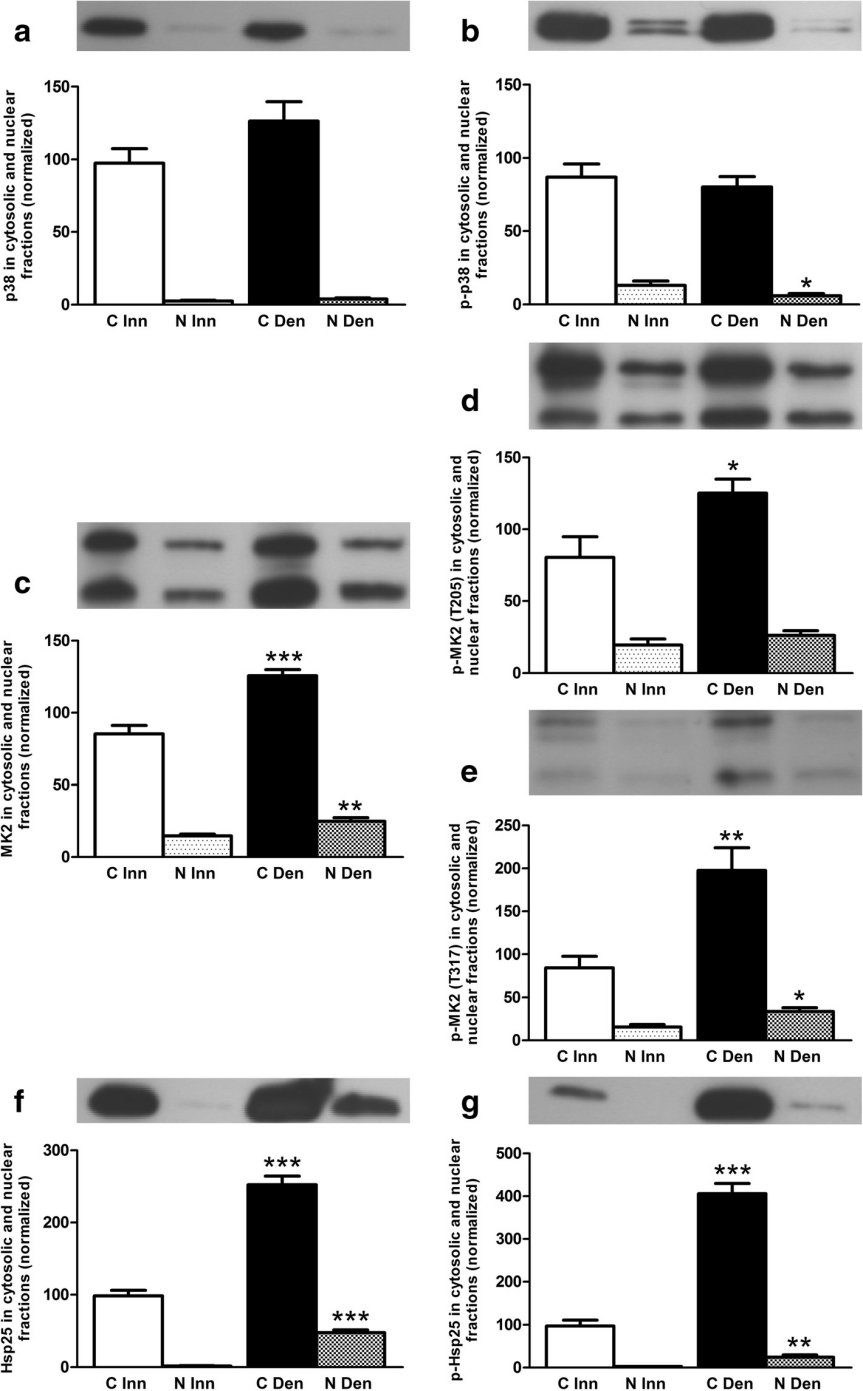
**Total and phosphorylated p38, MK2 and Hsp25 in cytosolic and nuclear fractions of 6-days denervated hypertrophic hemidiaphragm muscle.** Total and phosphorylated p38 **(a, b)**, MK2 **(c-e)** and Hsp25 **(f, g)** in cytosolic (C) and nuclear (N) fractions of 6-days denervated (Den) hypertrophic hemidiaphragm muscle compared to innervated (Inn) controls. Representative images of Western blots are shown together with densitometric quantifications. The labeling of the histograms also applies to the lanes in the Western blot images above the histograms. All bands shown on the Western blot images were analyzed together. One innervated cytosolic sample was loaded onto all gels as a reference. All samples were measured relative to this reference. The data were normalized so that the sum of cytosolic and nuclear signals in innervated muscles will give a mean value of 100.0. Mean values ± standard error of the mean. Statistical comparisons were made between cytosolic fractions of denervated versus innervated muscles and between nuclear fractions of denervated versus innervated muscles. *p < 0.05, **p < 0.01, ***p < 0.001, n = 8 denervated hemidiaphragm muscles and 8 innervated control hemidiaphragms from separate animals. Each muscle was fractionated into a cytosolic and a nuclear fraction.

In innervated as well as in 6-days denervated hypertrophic hemidiaphragm total and phosphorylated p38, MK2 and Hsp25 proteins were mainly present in cytosolic fractions. Total p38 protein expression was unchanged following denervation whereas the expression of total MK2 and Hsp25 were increased about 0.5-fold and 1.6-fold, respectively, in cytosolic fractions. Phosphorylated MK2 (T205 and T317) and Hsp25 were increased about 0.6-fold, 1.3-fold and 3.2 fold, respectively, in cytosolic fractions (Figure 2).

The mean expression levels of total p38 protein in cytosolic and nuclear fractions (Figure 2a) were 97.4 ± 10.1 arbitrary units and 2.6 ± 0.5 arbitrary units in innervated muscles compared to 126.4 ± 13.4 arbitrary units and 4.0 ± 0.7 arbitrary units in denervated muscles. The mean expression levels of phosphorylated p38 in cytosolic and nuclear fractions (Figure 2b) were 86.9 ± 9.1 arbitrary units and 13.1 ± 2.9 arbitrary units in innervated muscles compared to 80.1 ± 7.2 arbitrary units and 5.9 ± 1.5 arbitrary units (p < 0.05, Mann Whitney test) in denervated muscles.

The mean expression levels of total MK2 protein in cytosolic and nuclear fractions (Figure 2c) were 85.3 ± 5.9 arbitrary units and 14.7 ± 1.3 arbitrary units in innervated muscles compared to 125.6 ± 4.3 arbitrary units (p < 0.001, Student’s t-test) and 24.8 ± 2.6 arbitrary units (p < 0.01, Student’s t-test) in denervated muscles. The mean expression levels of phosphorylated MK2 (T205) in cytosolic and nuclear fractions (Figure 2d) were 80.5 ± 13.4 arbitrary units and 19.5 ± 4.3 arbitrary units in innervated muscles compared to 125.2 ± 9.8 arbitrary units (p < 0.05, Student’s t-test) and 26.3 ± 3.3 arbitrary units in denervated muscles. The mean expression levels of phosphorylated MK2 (T317) in cytosolic and nuclear fractions (Figure 2e) were 84.5 ± 13.1 arbitrary units and 15.5 ± 3.0 arbitrary units in innervated muscles compared to 197.6 ± 26.5 arbitrary units (p < 0.01, Student’s t-test) and 33.6 ± 4.4 arbitrary units (p < 0.05, Mann Whitney test) in denervated muscles.

The mean expression levels of total Hsp25 protein in the cytosolic and nuclear fractions (Figure 2f) were 98.3 ± 8.0 arbitrary units and 1.7 ± 0.3 arbitrary units in innervated muscles compared to 252.5 ± 11.8 arbitrary units (p < 0.001, Mann-Whitney test) and 47.8 ± 3.9 arbitrary units (p < 0.001, Student’s t-test) in denervated muscles. The mean expression levels of phosphorylated Hsp25 in cytosolic and nuclear fractions (Figure 2g) were 97.5 ± 13.5 arbitrary units and 2.6 ± 0.3 arbitrary units in innervated muscles compared to 406.2 ± 23.7 arbitrary units (p < 0.001, Student’s t-test) and 24.3 ± 5.5 arbitrary units (p < 0.01, Student’s t-test) in denervated muscles.

### Protein expression in nuclear and cytosolic fractions of 6-days denervated atrophic anterior tibial muscle

**Figure 3:**
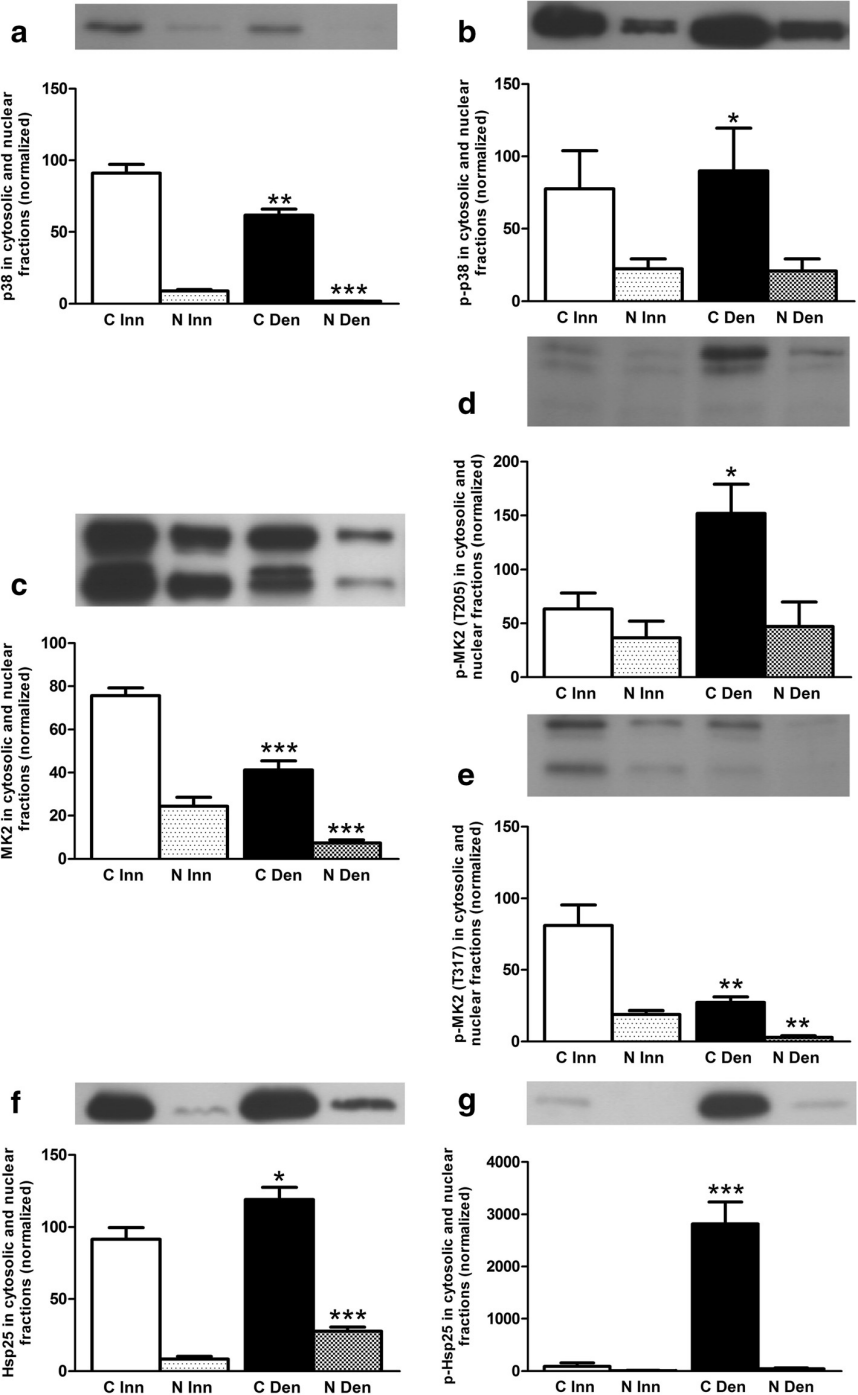
**Total and phosphorylated p38, MK2 and Hsp25 in cytosolic and nuclear fractions of 6-days denervated atrophic anterior tibial muscle.** Total and phosphorylated p38 **(a, b)**, MK2 **(c-e)** and Hsp25 **(f, g)** in cytosolic (C) and nuclear (N) fractions of 6-days denervated (Den) atrophic anterior tibial muscle compared to innervated (Inn) controls. Representative images of Western blots are shown together with densitometric quantifications. The labeling of the histograms also applies to the lanes in the Western blot images above the histograms. All bands shown on the Western blot images were analyzed together. One innervated cytosolic sample was loaded onto all gels as a reference. All samples were measured relative to this reference. The data were normalized so that the sum of cytosolic and nuclear signals in innervated muscles will give a mean value of 100.0. Mean values ± standard error of the mean. Statistical comparisons were made between cytosolic fractions of denervated versus innervated muscles and between nuclear fractions of denervated versus innervated muscles. *p < 0.05, **p < 0.01, ***p < 0.001, n = 8 denervated anterior tibial muscles and 8 contralateral innervated control muscles. Each muscle was fractionated into a cytosolic and a nuclear fraction.

In innervated as well as in 6-days denervated atrophic anterior tibial muscle total and phosphorylated p38, MK2 and Hsp25 proteins were mainly present in cytosolic fractions. Expression of total p38 and MK2 proteins were decreased about 0.3-fold and 0.5-fold, respectively, in cytosolic fractions of 6-days denervated muscle whereas the expression of Hsp25 was increased about 0.3-fold. Phosphorylated p38, MK2 (T205) and Hsp25 were increased about 0.2-fold, 1.4-fold and 30-fold, respectively, whereas the expression of phosphorylated MK2 (T317) decreased about 0.7-fold in cytosolic fractions following denervation (Figure 3).

The mean expression levels of total p38 protein in cytosolic and nuclear fractions (Figure 3a) were 91.0 ± 6.1 arbitrary units and 9.0 ± 0.9 arbitrary units in innervated muscles compared to 61.7 ± 4.3 arbitrary units (p < 0.01, Student’s paired t-test) and 1.8 ± 0.2 arbitrary units (p < 0.001, Student’s paired t-test) in denervated muscles. The mean expression levels of phosphorylated p38 in cytosolic and nuclear fractions (Figure 3b) were 77.5 ± 26.4 arbitrary units and 22.5 ± 6.9 arbitrary units in innervated muscles compared to 89.9 ± 29.7 arbitrary units (p < 0.05, Student’s paired t-test) and 20.9 ± 8.4 arbitrary units in denervated muscles.

The mean expression levels of total MK2 protein in cytosolic and nuclear fractions (Figure 3c) were 75.6 ± 3.6 arbitrary units and 24.4 ± 4.1 arbitrary units in innervated muscles compared to 41.2 ± 4.2 arbitrary units (p < 0.001, Student’s paired t-test) and 7.3 ± 1.4 arbitrary units (p < 0.001, Student’s paired t-test) in denervated muscles. The mean expression levels of phosphorylated MK2 (T205) in cytosolic and nuclear fractions (Figure 3d) were 63.4 ± 14.9 arbitrary units and 36.6 ± 15.5 arbitrary units in innervated muscles compared to 151.9 ± 27.1 arbitrary units (p < 0.05, Student’s paired t-test) and 47.0 ± 22.9 arbitrary units in denervated muscles. The mean expression levels of phosphorylated MK2 (T317) in cytosolic and nuclear fractions (Figure 3e) were 81.1 ± 14.3 arbitrary units and 18.9 ± 2.6 arbitrary units in innervated muscles compared to 27.2 ± 3.8 arbitrary units (p < 0.01, Student’s paired t-test) and 2.9 ± 1.2 arbitrary units (p < 0.01, Wilcoxon matched pairs test) in denervated muscles.

The mean expression levels of total Hsp25 protein in cytosolic and nuclear fractions (Figure 3f) were 91.6 ± 8.1 arbitrary units and 8.4 ± 1.8 arbitrary units in innervated muscles compared to 119.2 ± 8.4 arbitrary units (p < 0.05, Wilcoxon matched pairs test) and 27.9 ± 2.9 arbitrary units (p < 0.001, Student’s t-test) in denervated muscles. The mean expression levels of phosphorylated Hsp25 in cytosolic and nuclear fractions (Figure 3g) were 90.2 ± 69.4 arbitrary units and 9.8 ± 4.6 arbitrary units in innervated muscles compared to 2818.0 ± 420.2 arbitrary units (p < 0.001, Student’s paired t-test) and 46.2 ± 16.5 arbitrary units in denervated muscles.

### Protein expression in 6-days denervated pooled atrophic gastrocnemius and soleus muscles

**Figure 4:**
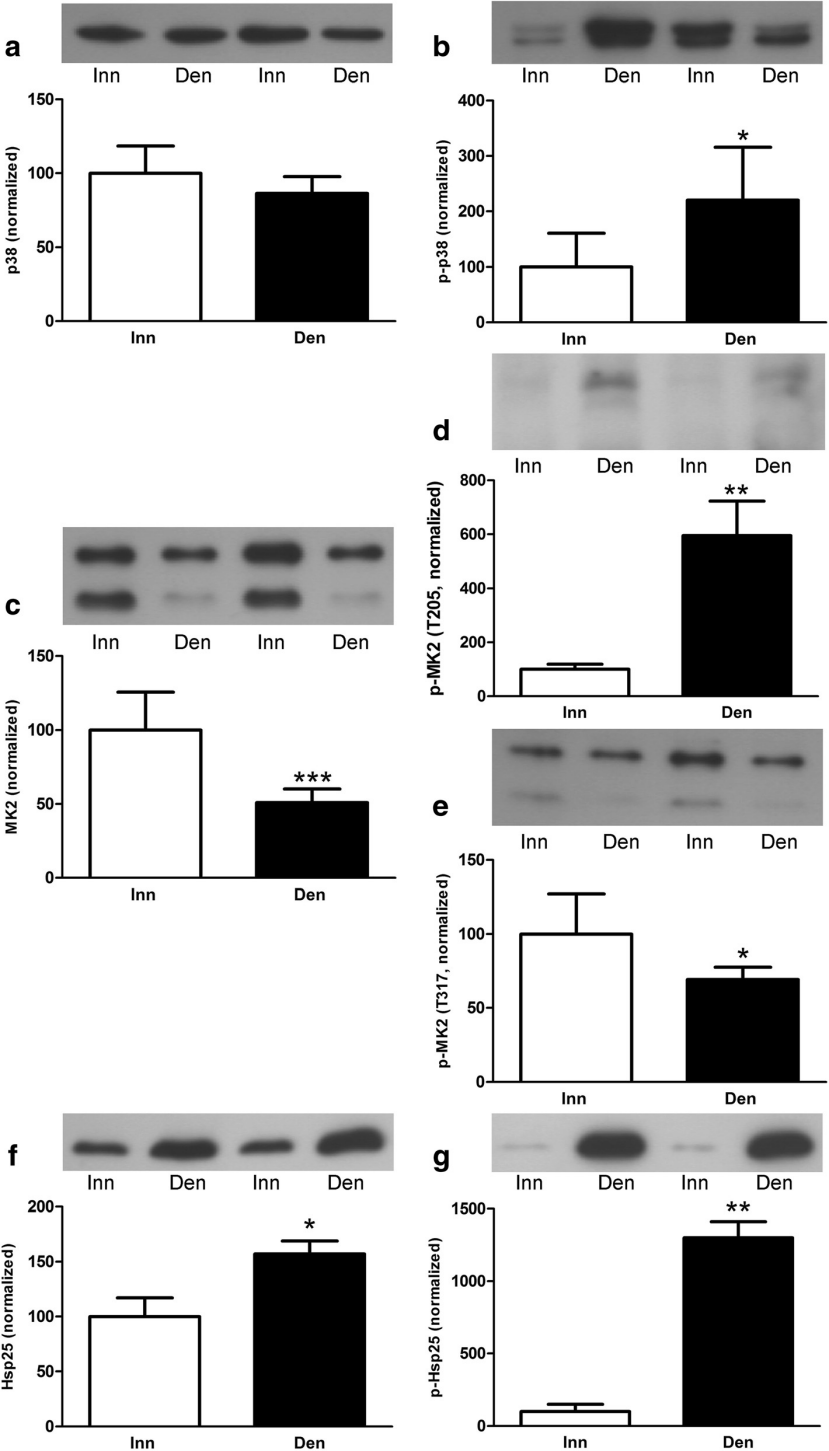
**Total and phosphorylated p38, MK2 and Hsp25 in 6-days denervated atrophic pooled gastrocnemius and soleus muscles.** Total and phosphorylated p38 **(a, b)**, MK2 **(c-e)** and Hsp25 **(f, g)** in 6-days denervated (Den) atrophic pooled gastrocnemius and soleus muscles compared to innervated (Inn) controls. Representative images of Western blots are shown together with densitometric quantifications. All bands shown on the Western blot images were analyzed together. One innervated sample was loaded onto all gels as a reference. All samples were measured relative to this reference. The data were normalized to give a mean value of 100.0 in innervated muscles. Mean values ± standard error of the mean. *p < 0.05, **p < 0.01, ***p < 0.001, n = 8 denervated pooled gastrocnemius and soleus muscles and 8 contralateral innervated control muscles.

In order to study hind-limb muscles that similar to hemidiaphragm muscle contain both type I and type II fibers, but like the anterior tibial muscle (no type I fibers, see Background) become atrophic following denervation, unfractionated homogenates of pooled gastrocnemius and soleus muscles were examined. In atrophic 6-days denervated pooled gastrocnemius and soleus muscles changes in the expression of total and phosphorylated p38, MK2 and Hsp25 were similar to those seen in denervated atrophic anterior tibial muscle. Expression of total p38 was unchanged whereas MK2 protein expression decreased about 0.5-fold and Hsp25 protein expression increased about 0.6-fold. Phosphorylated p38, MK2 (T205) and Hsp25 increased about 1.2-fold, 1.3-fold and 12-fold, respectively, whereas phosphorylated MK2 (T317) decreased about 0.3-fold following denervation (Figure 4).

The mean expression level of total p38 protein (Figure 4a) was 86.3 ± 4.0 arbitrary units in denervated muscles compared to 100.0 ± 6.5 in innervated muscles. The mean expression level of phosphorylated p38 (Figure 4b) was 220.1 ± 33.8 arbitrary units in denervated muscles compared to 100.0 ± 21.4 arbitrary units in innervated muscles (p < 0.05, Student’s t-test).

The mean expression level of total MK2 protein (Figure 4c) was 51.0 ± 3.3 arbitrary units in denervated muscles compared to 100.0 ± 9.1 arbitrary units in innervated muscles (p < 0.001, Student’s paired t-test). The mean expression level of phosphorylated MK2 (T205) (Figure 4d) was 226.5 ± 53.2 arbitrary units in denervated muscles compared to 100.0 ± 15.4 arbitrary units in innervated muscles (p < 0.05, Student’s t-test). The mean expression level of phosphorylated MK2 (T317) (Figure 4e) was 69.2 ± 3.0 arbitrary units in denervated muscles compared to 100.0 ± 9.6 arbitrary units in innervated muscles (p < 0.05, Student’s t-test).

The mean expression level of total Hsp25 protein (Figure 4f) was 157.0 ± 11.8 arbitrary units in denervated muscles compared to 100.0 ± 17.1 arbitrary units in innervated muscle (p < 0.05, Wilcoxon matched pairs test). The mean expression level of phosphorylated Hsp25 (Figure 4g) was 1301.0 ± 111.2 arbitrary units in denervated muscles compared to 100.0 ± 51.0 arbitrary units in innervated muscles (p < 0.01, Wilcoxon matched pairs test).

### Hsp70 protein expression in innervated and 6-days denervated muscles

**Figure 5:**
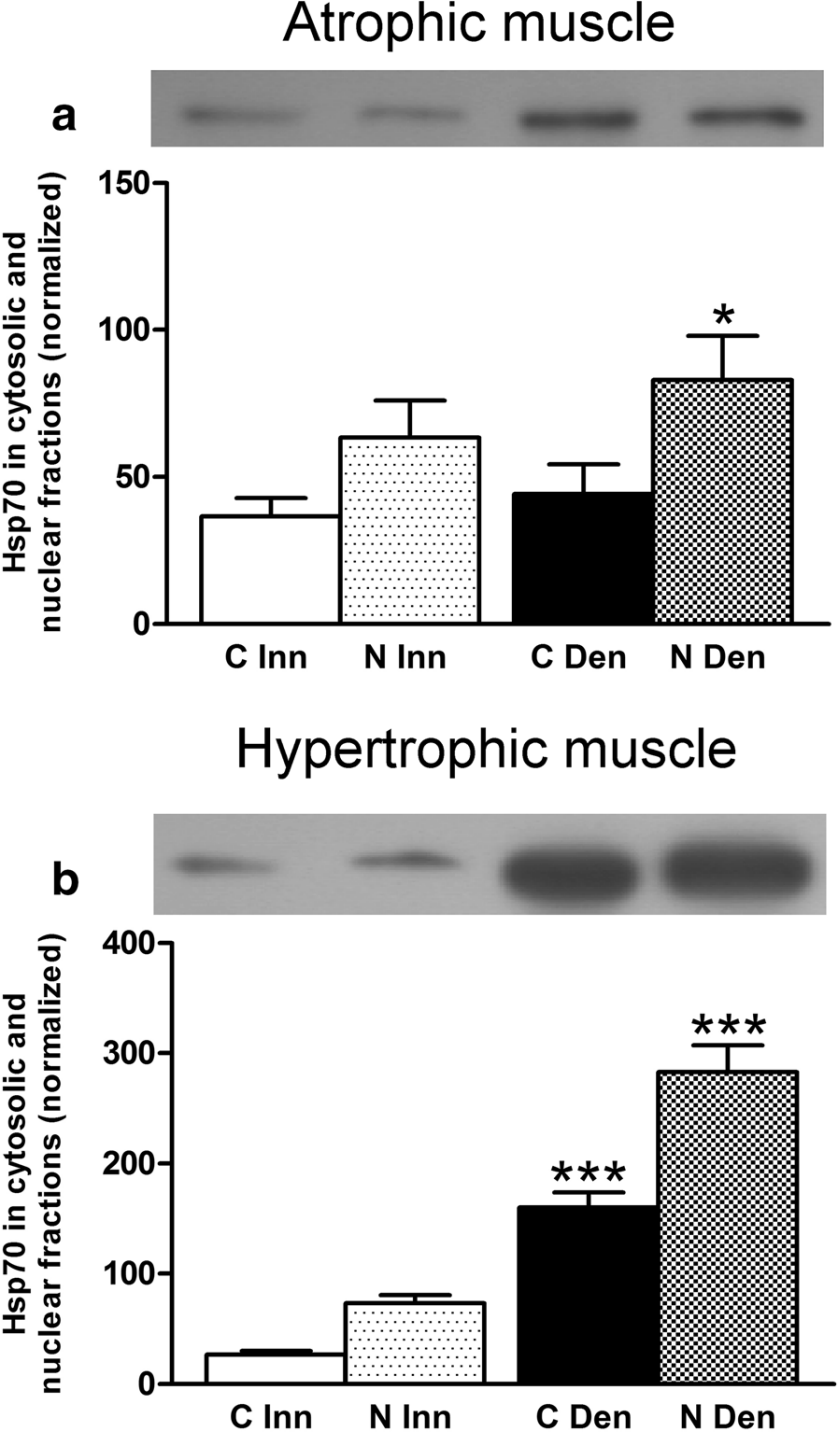
**Hsp70 protein expression in cytosolic and nuclear fractions of 6-days denervated atrophic and 6-days denervated hypertrophic muscle.** Hsp70 expression in cytosolic (C) and nuclear (N) fractions of 6-days denervated (Den) atrophic anterior tibial muscle **(a)** and in 6-days denervated hypertrophic hemidiaphragm muscle **(b)** compared to innervated (Inn) controls. Representative images of Western blots are shown together with densitometric quantifications. The labeling of the histograms also applies to the lanes in the Western blot images above the histograms. One innervated cytosolic sample was loaded onto all gels as a reference. All samples were measured relative to this reference. The data were normalized so that the sum of cytosolic and nuclear signals in innervated muscles will give a mean value of 100.0. Mean values ± standard error of the mean. Statistical comparisons were made between cytosolic fractions of denervated versus innervated muscles and between nuclear fractions of denervated versus innervated muscles. *p < 0.05, ***p < 0.001, n = 8 denervated anterior tibial muscles and 8 contralateral innervated control muscles **(a)** and 8 denervated hemidiaphragm muscles and 8 innervated control hemidiaphragms from separate animals **(b)**. Each muscle was fractionated into a cytosolic and a nuclear fraction.

**Figure 6:**
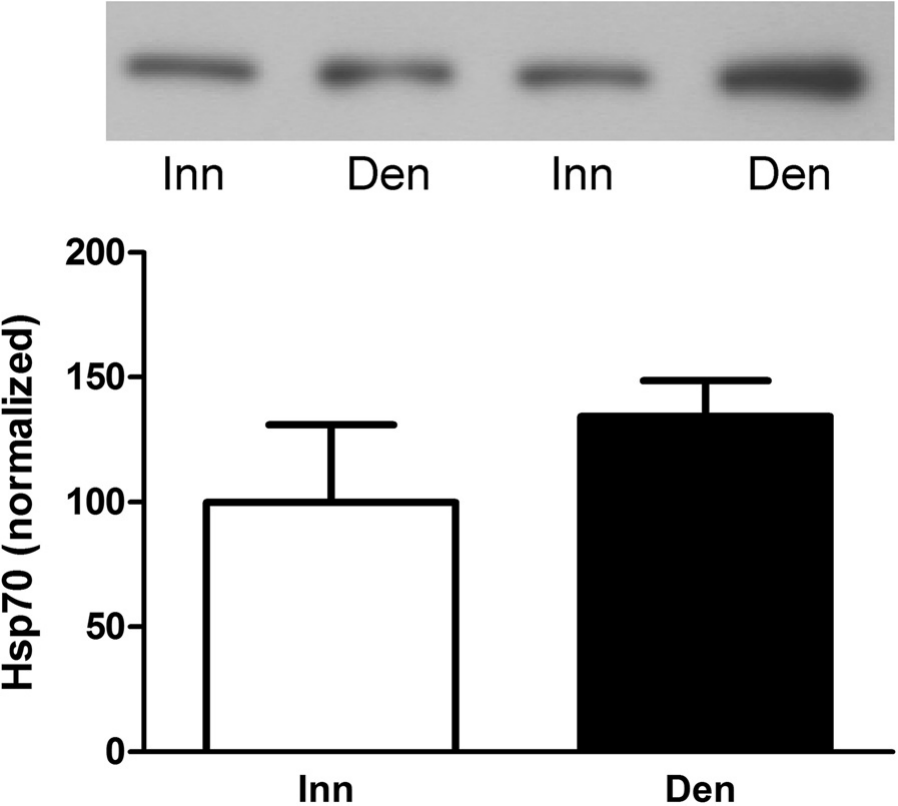
**Hsp70 protein expression in 6-days denervated atrophic pooled gastrocnemius and soleus muscles.** Hsp70 expression in 6-days denervated (Den) atrophic pooled gastrocnemius and soleus muscles compared to innervated (Inn) controls. Representative images of Western blots are shown together with densitometric quantifications. One innervated sample was loaded onto all gels as a reference. All samples were measured relative to this reference. The data were normalized to give a mean value of 100.0 in innervated muscles. Mean values ± standard error of the mean, n = 8 denervated pooled gastrocnemius and soleus muscles and 8 contralateral innervated control muscles.

Hsp70 expression was studied since it has recently been reported to play a role in the phosphorylation of MK2 by p38 (see Background). Unlike the other proteins studied Hsp70 was more abundant in nuclear than in cytosolic fractions of innervated as well as 6-days denervated muscles. In atrophic 6-days denervated anterior tibial muscles the expression of Hsp70 was slightly increased (about 0.3-fold) in the nuclear fraction compared to innervated controls. In 6-days denervated hypertrophic hemidiaphragm the expression of Hsp70 was increased about 5-fold and 2.9-fold in cytosolic and nuclear fractions, respectively (Figure 5). There was no statistically significant change in Hsp70 expression in atrophic pooled 6-days denervated gastrocnemius and soleus muscles compared to innervated controls (Figure 6).

In anterior tibial muscle the mean expression levels of total Hsp70 protein in cytosolic and nuclear fractions (Figure 5a) were 36.6 ± 6.2 arbitrary units and 63.4 ± 12.6 arbitrary units in innervated muscles compared to 44.2 ± 10.0 arbitrary units and 83.1 ± 14.9 arbitrary units (p < 0.05, Student’s t-test) in denervated atrophic muscles.

In hemidiaphragm muscle the mean expression levels of total Hsp70 protein in cytosolic and nuclear fractions (Figure 5b) were 26.8 ± 3.5 arbitrary units and 73.2 ± 7.3 arbitrary units in innervated muscles compared to 160.1 ± 13.8 arbitrary units (p < 0.001, Student’s t-test) and 282.9 ± 24.3 arbitrary units (p < 0.001, Student’s t-test) in denervated hypertrophic muscles.

In 6-days denervated atrophic pooled gastrocnemius and soleus muscles the mean expression level of total Hsp70 protein (Figure 6) was 134.4 ± 14.2 arbitrary units compared to 100.0 ± 31.0 arbitrary units in innervated muscles.

## Discussion

The present study confirms previous observations [48] of increased MK2 T205 phosphorylation in denervated skeletal muscle and a differential response of MK2 T317 phosphorylation to denervation in hemidiaphragm (hypertrophic, increased T317 phosphorylation) and anterior tibial muscle (atrophic, decreased T317 phosphorylation). The present study also shows increased MK2 T205 phosphorylation and decreased T317 phosphorylation in denervated atrophic pooled gastrocnemius and soleus muscles. Since the T317 phosphorylation is suggested to be important for nuclear export of MK2 (see Background), possibly in complex with p38, the previous study [48] suggested that in hypertrophic denervated muscle MK2 and/or p38 may be localized mainly in the cytoplasm whereas in denervated atrophic muscle they may be localized in the nucleus to a larger extent. In the present study on separated cytosolic and nuclear fractions no evidence was obtained for a preferential nuclear localization of MK2 and/or p38 in denervated atrophic anterior tibial muscle. The present study shows that both p38 and MK2 are mainly localized in the cytosolic fraction in innervated as well as in denervated muscle. Successful separation of nuclear and cytosolic fractions was confirmed using Western blots for Lamin A/C and SOD-1, respectively.

The differential effect of denervation on T317 phosphorylation of MK2 in hemidiaphragm muscle and hind-limb muscle (anterior tibial muscle and pooled gastrocnemius and soleus muscles) was not reflected in p38 phosphorylation. Thus, in denervated atrophic pooled gastrocnemius and soleus muscles p38 phosphorylation was increased (Figure 4b) whereas MK2 T317 phosphorylation was decreased (Figure 4e). Similarly, in denervated atrophic anterior tibial muscle p38 phosphorylation was slightly increased in cytosolic fractions (Figure 3b) whereas MK2 T317 phosphorylation was markedly decreased in cytosolic as well as in nuclear fractions (Figure 3e). In denervated hypertrophic hemidiaphragm p38 phosphorylation was slightly decreased in nuclear fractions (Figure 2b) whereas MK2 T317 phosphorylation was increased in nuclear as well as cytosolic fractions (Figure 2e). This suggests that factors other than phosphorylated p38 are important for the T317 phosphorylation of MK2. One such factor may be Hsp70 that has recently been reported to play a role in the phosphorylation of MK2 by p38 [34]. In the present study a substantial increase in Hsp70 was observed in cytosolic and nuclear fractions of hypertrophic 6-days denervated hemidiaphragm whereas in 6-days denervated atrophic anterior tibial muscle only a small increase in Hsp70 was seen in the nuclear fraction. No statistically significant increase in Hsp70 was observed in 6-days denervated pooled gastrocnemius and soleus muscles. Previous studies [52] have, however, reported some increase in Hsp70 in 14-days denervated gastrocnemius muscle and 15-days denervated anterior tibial muscle [53]. In other models of muscle atrophy and hypertrophy Hsp70 has been shown to be down-regulated in atrophic conditions such as immobilization [38] and hind-limb unloading [35-37] whereas it is up-regulated in hypertrophic conditions such as functional overload [39-44]. Overexpression of Hsp70 has also been shown to prevent skeletal muscle atrophy caused by immobilization [32,33] and to improve recovery [54]. Interestingly, in various models of skeletal muscle atrophy Hsp70 content was found to correlate with muscle weight [55]. A similar correlation between muscle weight and T317 phosphorylation of MK2 was observed in denervated atrophic and hypertrophic muscle [48]. In future studies it would be interesting to see if overexpression or knockdown of Hsp70 affects MK2 T317 phosphorylation in skeletal muscle.

The discrepancies between p38 phosphorylation and MK2 T317 phosphorylation may also be due to different isoforms of p38 responding differently to denervation. The antibodies used in the present study do not differentiate between the different p38 isoforms and while all p38 isoforms appear to be expressed to some extent in skeletal muscle [56-59] p38γ and p38δ have very low kinase activity towards the substrate MK2 [56,58-60]. The isoform p38γ is required for mitochondrial biogenesis and angiogenesis in response to endurance exercise [61] and is preferentially activated in slow muscle [62] whereas overexpression of p38β up-regulates the E3 ubiquitin ligase atrogin1/MAFbx and causes a loss of muscle mass [63]. The isoform p38α has an essential role in myogenesis [64].

Following denervation the level of Hsp25 was increased in all muscles studied, as was the level of phosphorylated Hsp25. Similar to the discordance between p38 phosphorylation and MK2 T317 phosphorylation, the level of MK2 T317 phosphorylation was not reflected in Hsp25 phosphorylation in atrophic muscle. In atrophic anterior tibial muscles as well as in atrophic pooled gastrocnemius and soleus muscles Hsp25 phosphorylation was increased (Figures 3g and 4g) despite decreased levels of MK2 phosphorylated at T317 (Figures 3e and 4e). This suggests that, at least in denervated atrophic hind-limb muscle, other factors in addition to MK2 are important for determining the phosphorylation level of Hsp25. A number of different kinases and/or phosphatases might be involved in this [65]. Previous studies have shown increased Hsp25/27 phosphorylation in hypertrophy caused by functional overload [13,30,39,66] and decreased phosphorylation in atrophic rat soleus muscle after 14 days of hind-limb unloading [30]. Overexpression of Hsp27 has also been shown to attenuate atrophy of the rat soleus muscle following immobilization [31]. Previous studies on denervated muscle have shown increased Hsp25/27 levels in rat extensor digitorum longus and plantaris muscles after 6-8 days of denervation [67,68] but decreased levels in rat soleus muscles 6 days after denervation [68]. In the present study on denervated mouse muscles increased levels of Hsp25 were found in all muscles studied 6 days after denervation (hemidiaphragm, anterior tibial and pooled gastrocnemius and soleus muscles). The discrepancy between the present study and the decreased levels of Hsp25/27 in denervated rat soleus muscle may be related to the pooling of muscles in the present study or species differences. In possible support of a species difference, normal innervated rat soleus muscle has been shown to contain considerably higher levels of Hsp25/27 than extensor digitorum longus muscle [68,69] and plantaris muscle [39]. In the mouse plantaris muscle, however, Hsp25/27 levels were found to be higher than in the rat plantaris muscle and no significant difference was observed between Hsp25/27 levels in mouse plantaris and soleus muscles [39]. Regarding phosphorylated Hsp25/27 a previous study on rat plantaris muscle did not find any increase in phosphorylated Hsp25/27 after 8 days of bilateral denervation [67]. This is in contrast to the results obtained in the present study showing increased Hsp25 phosphorylation in all denervated mouse muscles studied 6 days after unilateral denervation. This apparent discrepancy may also be related to species differences or to differences in experimental design such as bilateral versus unilateral denervation.

## Conclusions

The present study confirms a differential response of MK2 T317 phosphorylation to denervation in hemidiaphragm (hypertrophic, increased T317 phosphorylation) and hind-limb muscle (atrophic, decreased T317 phosphorylation) and suggests that factors other than p38 may be important for this differential response, e.g. Hsp70. The study further shows that, despite the differential response of MK2 T317 phosphorylation to denervation in the different muscles, Hsp25 phosphorylation was increased in all denervated muscles studied indicating a role for other kinases and/or phosphatases in the regulation of Hsp25 phosphorylation in denervated muscle.

## Methods

### Animals and muscles

All experiments were performed on adult male NMRI mice (NOVA-SCB, Sollentuna, Sweden). Before surgery the animals were anaesthetized by inhalation of isoflurane. Denervation of either the left hind-limb or the left hemidiaphragm was performed by sectioning and removing a few mm of either the sciatic nerve or the phrenic nerve as described previously [70]. While anaesthetized the animals received a subcutaneous injection of buprenorphine (50 μg/kg) for post-operative analgesia. Before and after surgery the mice were kept in cages with environment enrichment and free access to a standard laboratory diet and tap water. Six days after denervation the mice were killed by cervical dislocation. Hind-limb muscles (either anterior tibial or gastrocnemius together with soleus) were rapidly dissected, weighed, frozen on dry ice and stored at -80°C. Innervated control hind-limb muscles (either anterior tibial or gastrocnemius together with soleus) were collected from the contralateral (right) leg of animals that were denervated by sectioning the left sciatic nerve. Muscles from the innervated leg were dissected first in every second animal and muscles from the denervated leg were dissected first in the remaining animals. For dissection of the hemidiaphragm muscle the diaphragm, attached to the rib cage, was quickly removed and placed in cold phosphate buffered saline (PBS) with calcium (2 mM). The left hemidiaphragm was then dissected under a dissecting microscope, blotted dry on filter paper, weighed, frozen on dry ice and stored at -80°C. Innervated left control hemidiaphragms were collected from separate animals that had received no surgery. The experimental manipulations have been approved by the Ethical Committee for Animal Experiments, Linköping, Sweden.

### Nuclear and cytosolic fractions

Mouse hemidiaphragm and anterior tibial muscles were used for nuclear and cytosolic protein extraction. The method used was slightly modified from [71]. The muscles were homogenized using an Ultra-Turrax homogenizer (Janke and Kunkel, Staufen, Germany) in 1 ml low salt lysis buffer (10 mM HEPES, 10 mM KCl, 1.5 mM MgCl_2_, 0.1 mM EDTA, 0.1 mM EGTA, 1 mM dithiothreitol (DTT), pH 7.9 with 1% Halt™ Protease and Phosphatase Inhibitor Cocktail from Thermo Scientific, Rockford, IL). The homogenized tissue was vortexed for 15 s, put on ice for 10 min, vortexed again for 15 s and centrifuged at 16,000 g for 15 s. The supernatant cytosolic extract was immediately frozen (-80°C) for subsequent analyses. The nuclear pellet was resuspended on ice in a high salt nuclear extraction buffer (20 mM HEPES, 420 mM NaCl, 1 mM EDTA, 1 mM EGTA, 1 mM DTT, 25% glycerol, pH 7.9 with 1% Halt™ Protease and Phosphatase Inhibitor Cocktail from Thermo Scientific, Rockford, IL) at a ratio of 4 μl of nuclear extraction buffer per mg muscle wet weight. Preparations were incubated on ice for 30 min and vortexed for 10 s every 5 min before being centrifuged at 16,000 g for 6 min. The supernatant nuclear extract was then removed and frozen (-80°C) for subsequent analyses. Protein concentrations for each fraction were obtained using the Bradford assay [72].

### Unfractionated muscle homogenates

Pooled gastrocnemius and soleus muscles were used for preparing protein extracts of unfractionated muscle homogenates. The muscles were homogenized in 2 ml of a buffer containing 100 mM Tris-HCl, pH 7.6, 150 mM NaCl, 1 mM EDTA, 1% NP-40, 0.1% sodium deoxycholate with 1% Halt™ Protease and Phosphatase Inhibitor Cocktail from Thermo Scientific (Rockford, IL). The homogenate was then centrifuged at 16,000 g for 10 min. The supernatant was recovered and the pellet was resuspended in 2 ml of buffer and re-centrifuged. The supernatants were combined and the protein concentration was determined using the Bradford assay [72].

### Western blots

Western blots were prepared essentially as described in [73]. Ten to forty μg protein were reduced, denaturated and electrophoretically separated on a 12% polyacrylamide gel with a 5.2% polyacrylamide stacking gel on top. Equal amounts of cytosolic and nuclear protein from innervated and denervated muscles were loaded onto the gels. Gels were electroblotted onto PVDF Plus membranes (Amersham Hybond-P, GE Healthcare, Buckinghamshire, England) and the membranes were blocked and then incubated with antibodies. Primary antibodies for detecting total Lamin A/C [2032], total p38 MAPK [9212], phospho-p38 MAPK (T180/Y182) [4511], total MK2 [3042], phospho-MK2 (T334, corresponds to T317 in the mouse) [3041], phospho-MK2 (T222, corresponds to T205 in the mouse) [3316], total Hsp27 (Hsp25 in the mouse) [2442], phospho-Hsp27 (S82, corresponds to S86 in mouse Hsp25) [2401] and Hsp70 (D69) [4876] were from Cell Signaling Technology (Beverly, CA). Primary antibody for detecting SOD-1 (C-17) [8637] was from Santa Cruz Biotechnology (Santa Cruz, CA). All primary antibodies were used at a dilution of 1/1,000-1/10,000. Antibodies were visualized with horseradish peroxidase conjugated secondary immunoglobulin diluted 1/1,000-1/2,000 (goat anti-rabbit IgG [P0448], rabbit anti-mouse [P0260] or rabbit anti-goat [P0449], (Dako, Glostrup, Denmark). Negative controls included membranes incubated in the absence of the primary antibodies. The bound immune complexes were detected using the ECL Plus Western blotting detection system and Hyperfilm ECL (Amersham International and Amersham Pharmacia Biotech, Buckinghamshire, England).

### Data analysis and statistics

The expression levels of total and phosphorylated proteins were studied semi-quantitatively using data from Western blots. Measured levels of total and phosphorylated proteins were expressed without normalization to any specific protein. No loading controls were used and any difference in protein quantifications, pipetting steps, protein transfers etc. are included in the variations of the data sets.

Image analysis was performed using the gel plotting macro of the program ImageJ (Rasband, W.S., ImageJ, US National Institutes of Health, Bethesda, MD, http://rsb.info.nih.gov/ij/, 1997-2007). Results were obtained in uncalibrated units.

MK2 from mouse migrates as two bands of sizes 54 and 45 kDa [74]. Both bands (sometimes doublets) were quantified and the summed signal was used as an estimate of the expression of total MK2 protein or different phosphorylated species.

For quantification of protein expression in nuclear and cytosolic fractions one of the cytosolic fractions from an innervated muscle was used as a reference sample and was included in all gels. All other samples were measured relative to this reference, the signal of which was set to 100.0. From the amount of protein loaded on gels in relation to the total amount of protein extracted in the nuclear and cytosolic fractions a total cytosolic and a total nuclear signal was calculated for whole muscles. For easy comparisons of the relative amounts of the different proteins/phosphorylations in nuclear and cytosolic fractions, the sum of the nuclear and cytosolic signals was finally normalized to 100.0 in innervated muscle.

For quantification of protein expression in total pooled gastrocnemius and soleus muscle homogenates one of the innervated muscle samples was used as a reference sample and was included in all gels. All other samples were measured relative to this reference, the signal of which was set to 100.0. In the final analysis all signals were again normalized in such a way that the average signal from innervated muscles became 100.0.

Data are presented as mean values ± standard error of the mean (SEM). Student’s t-test was used for statistical comparisons of normally distributed data (D’Agostino-Pearson omnibus K2 normality test). Statistical significance for data that was not normally distributed was determined using the Wilcoxon matched pairs test (anterior tibial muscle) and Mann-Whitney test (hemidiaphragm muscles).

## Competing interests

The authors declare no competing interests.

## Authors’ contributions

The work presented here was carried out in collaboration between all authors. KE designed the study and carried out the studies on cytosolic and nuclear fractions, most of the protein expression studies, statistical analyses and drafted the manuscript. AKF carried out the studies on total and phosphorylated p38, MK2 and Hsp25 in 6-days denervated atrophic pooled gastrocnemius and soleus muscles. MN and ST conceived of the study, participated in the design, statistical analyses and drafting of the manuscript. All authors have read and approved the final manuscript.
